# An Evaluation of the Effects of Pepper (*Zanthoxylum bungeanum Maxim.*) Leaf Extract on the Physiochemical Properties and Water Distribution of Chinese Cured Meat (Larou) During Storage

**DOI:** 10.3390/foods13233972

**Published:** 2024-12-09

**Authors:** Shengming Zhao, Mengke Li, Mengran Hei, Yanyan Zhao, Jingjun Li, Zhuangli Kang, Hanjun Ma, Guoyuan Xiong

**Affiliations:** 1 School of Food Engineering, Anhui Science and Technology University, No.9 Donghua Road, Fengyang 233100, China; zhaoshengming2008@126.com (S.Z.);; 2 School of Food Science and Technology, Henan Institute of Science and Technology, No.90 Hua lan Street, Xinxiang 453003, China; 3 Anhui Province Key Laboratory of Functional Agriculture and Functional Food, Anhui Science and Technology University, Chuzhou 239000, China; 4 School of Tourism and Cuisine, Yangzhou University, Yangzhou 225127, China

**Keywords:** ZL extract, larou, quality change, water distribution, protein structure

## Abstract

In this study, pepper (*Zanthoxylum bungeanum Maxim.*) leaf (ZL) extract was added to larou to investigate the improvement in the quality of physicochemical properties, texture, water distribution, and microorganism growth during storage for 20 days. Based on the results, the addition of ZL extract significantly retarded the increase in cooking loss, TBARS value, hardness, and microorganism growth. Moreover, the addition of ZL extract decreased the pH value, lightness, and microorganism counts, and increased the moisture content, total soluble protein content, a* value, b* value, and chewiness. The LF-NMR results showed that the addition of ZL extract shortened the T_2_ relaxation time and boosted the proportion of immobilized water, facilitating the validation of the improvement in water retention of larou during storage. The FT-IR results indicated that the addition of ZL extract influenced the protein secondary structure by inducing the conversion of α-helices to β-sheet structures. Accordingly, ZL extract has the potential to serve as a natural antioxidant, effectively helping to ameliorate the quality properties of cured meat products during storage.

## 1. Introduction

Larou (Chinese cured bacon) is a traditional cured meat product in China, favored for its savory taste, nutritional benefits, and convenience in terms of carrying and transportation [1]. There are various types of larou which are widely distributed in China, including the famous Cantonese style, the Hunan style, the Yunan style, and Sichuan-style larou. The processing and storage of larou involves protein degradation and fat oxidation, resulting in the production of volatile compounds such as alcohols, aldehydes, and aromatic hydrocarbons that contribute to its unique flavor profile [2,3]. However, it has been reported that the excessive lipid and protein oxidation, as well as microbial contamination, are prone to occur during storage, leading to decreased color, texture, and nutritional components, resulting in nutrient loss and quality deterioration [4]. Therefore, the major challenge for dry-cured meat processing and storage is how to prevent and control excessive oxidation to maintain product quality.

Synthetic antioxidants such as butylated hydroxyanisole (BHA), butylated hydroxytoluene (BHT), and tert-butylhydroquinone (TBHQ) are widely utilized to inhibit excessive oxidation during larou processing and storage. Despite their benefits, there are concerns regarding the potential toxicity and carcinogenicity of synthetic antioxidants in humans [5,6]. Thus, with increasing awareness of health among individuals, natural plant extracts, predominantly derived from vegetables, fruits, and traditional Chinese medicinal herbs, have garnered burgeoning attention from consumers as natural and safe antioxidants [7]. An increasing body of literature indicates that plant extracts have been used as alternative natural antioxidants to prevent lipid and protein oxidation, inhibit microorganism growth, and improve the quality of cured meat in virtue of their antioxidant activities [8,9]. Therefore, natural plant extracts exhibit extensive application potential in the processing of meat products.

Chinese red pepper (*Zanthoxylum bungeanum Maxim*.) is one of the important traditional spices in China, and its leaves are generally considered as agricultural waste with a low utilization rate. However, recent studies have indicated that pepper leaves are rich in various natural active components, including polyphenols, polysaccharides, and alkaloids, which shows their distinct antibacterial and antioxidant activities [10,11,12,13]. Notably, phenolic compounds in natural plant extracts have been proven to significantly inhibit the excessive oxidation of fats and proteins during the processing of meat products [3,6]. Research by Li, Liu, Wang, Liu, and Peng [14] and Li, Wang, Li, and Peng [15] has found that the pepper leaf extract contained higher amounts of chlorogenic acid, hyperin, and quercetin, which can effectively alleviate the lipid oxidation of salted silver carp. Our previous study has also shown that pepper leaf extract exhibited a remarkable inhibitory effect on fat and protein oxidation during cured meat processing [16]. However, to the best of our knowledge, no research has been carried out on the applications of leaf extract on the quality changes in cured meat during storage. Thus, this study primarily investigates the impact of pepper leaf extract on the quality of traditional Chinese larou during storage by assessing variations in physicochemical properties, texture, water migration, and protein secondary structure.

## 2. Materials and Methods

### 2.1. Materials

The dry pepper (*Zanthoxylum bungeanum Maxim*.) leaves (ZL) were obtained from the meat science laboratory of the Henan Institute of Science and Technology and smashed by a grinder to 50 mesh. ZL extracts were obtained by ultrasonic-assisted ethanol extraction (SB-4200 DTD, Scientz, Ningbo, China) on the basis of our previous study [16]. Fresh lean pork was purchased from the local supermarket (Xinxiang, China). Sodium chloride, potassium chloride, sodium nitrite, thiobarbituric acid, trichloroacetic acid, and potassium bromide (KBr) were purchased from Kemio Co., Ltd. (Tianjin, China). All reagents used are analytical grade, except for KBr (spectral grade).

### 2.2. Manufactures of Larou

The preparation of larou samples was conducted based on the method described in our previous study [16]. Fresh lean pork without skin was sectioned into pieces of uniform weight (120 ± 5 g) and marinated for 2 h with 4.0% salt (*w*/*w*) and 0.08 g/kg sodium nitrite (*w*/*w*). The samples were randomly assigned into three groups (25 pieces in each group) and treated with ZL extract (*w*/*w*) as follows: (1) control group (without ZL extract); (2) 0.02% ZL extract group; and (3) 0.04% ZL extract group. The marinating process was carried out at 4 °C for two days. During this process, the samples were flipped every 12 h to ensure even marination. Subsequently, the samples were transferred to a constant temperature incubator at 27 °C for 20 days of storage and evaluated at 0, 5, 10, 15 and 20 days, respectively.

### 2.3. Moisture Content

The moisture content of larou was determined using the direct drying method of AOAC [17]. Briefly, the larou samples (5 g) were dried at 105 °C with a constant weight. The moisture content was calculated as follows:

Moisture content (%) = (W_0_ − W_1_)/W_0_ × 100%

where W_0_ is the weight (g) of the sample before drying; and W_1_ is the weight (g) of the sample after drying.

### 2.4. pH

The pH of larou was measured following the previous study [18] with some modifications. The larou sample (1 g) was minced and mixed with 10 mL of a 7.5% potassium chloride solution. The mixtures were homogenized at 10,000 rpm for 30 s (T-25 Ultra Turrax, IKA, Staufen, Germany), and the pH value of the sample was recorded using a digital pH meter (Seven Compact S220; Mettler, Zurich, Switzerland).

### 2.5. Total Soluble Protein

The total soluble protein of larou was determined based on a previously used method [19]. First, the larou sample (4 g) was mixed with 400 mL of phosphate buffer (pH 7.0). The mixtures were then centrifuged for 30 min (4 °C, 8000× *g*) after homogenizing at 10,000 rpm for 30 s in an ice bath. The collected supernatants were used to measure the content of the total soluble protein by the Biuret method.

### 2.6. Cooking Loss

The cooking loss of larou was determined using the method described by Angel-Ángel-Rendón, Filomena-Ambrosio, Cordon-Díaz, Benítez-Sastoque, and Sotelo-Díaz [20]. The samples (20 g), wrapped with a retort pouch, were heated in a water bath at 85 °C for 20 min. Then, after being cooled to 25 °C, the moisture on the surface of the samples was blotted with absorbent paper and they were reweighed accurately. The cooking loss was evaluated as follows:

Cooking loss (%) = (W_0_ − W_1_)/W_0_ × 100%

where W_0_ is the weight (g) of the sample before cooking; and W_1_ is the weight (g) of the sample after cooking.

### 2.7. Texture

The texture (hardness, springiness, cohesiveness and chewiness) of larou was analyzed as described by Xu et al. [8] using a texture analyzer (TA-XT plus, Stable Micro Systems, Surrey, UK) with the p36R probe in TPA mode. The testing parameters were as follows: compression ratio of 40%, test speed of 0.5 mm/s, pre-test speed of 1 mm/s, post-test speed of 1 mm/s, trigger force of 5 g, and interval time of 5 s.

### 2.8. Instrumental Color

The surface color of the larou sample was determined by a Chroma meter CR-400 (Konica Minolta, Tokyo, Japan) with a Luminant of D 65. The chroma meter was calibrated using a white plate and the values of L* (lightness), a* (redness) and b* (yellowness) were recorded. Five determinations were made in each portion of larou [21].

### 2.9. Thiobarbituric Acid Reactive Substances (TBARS)

The TBARS values of larou were determined using the method described by Wu et al. [2] with slight modifications. In brief, each minced larou sample (5 g) was homogenized with 50 mL of trichloroacetic acid solution (7.5%) and vibrated at 50 °C for 30 min. Then, after being cooled to 25 °C, the homogenates were filtered through filter paper and the filtrates were harvested. The 5 mL of filtrates were reacted with 5 mL of thiobarbituric acid (TBA) solution in a 90 °C water bath for 30 min. The trichloroacetic acid solution was used as a control. After centrifugation for 10 min (4 °C, 3000× *g*), the absorbance of the sample supernatants was recorded at 532 nm using a UV-1800 spectrophotometer (Shimadzu Corporation, Kyoto, Japan). The TBARS values were calculated according to a standard malondialdehyde (MDA) curve (y = 1.1096x + 0.0118) and expressed as mg MDA/kg larou.

### 2.10. Total Viable Counts (TVC) of Bacteria

Larou samples (25 g) were blended with 225 mL of sterile saline solution (0.09 g/kg NaCl) for 2 min. Then, 1 mL of each sample solution was spread on plate count agar (Aobox Co., Ltd., Beijing, China) following the 10-fold dilution approach, and incubated at 37 °C for 48 h to assess TVC. The result was expressed as log_10_ CFU/g [5].

### 2.11. Low-Field Nuclear Magnetic Resonance (LF-NMR)

The larou samples were sliced into homogeneous cubes (1.5 cm × 1.5 cm × 2 cm) and placed in a low-field NMR glass tube, which was measured in a low-field NMR analyzer (Model PQ001, Niumag Electric Corporation, Shanghai, China) with a resonance frequency of 22.6 MHz. The spin–spin relaxation time (T_2_) and its corresponding water cluster distribution ratio (PT_2_) were measured using the CPMG sequence with a τ value of 350 μs [22].

### 2.12. Fourier Transform Infrared (FT-IR) Spectroscopy

According to our previous method [22], the vacuum-freeze-dried larou samples were ground and homogeneously mixed with KBr at a ratio of 1:100, followed by pressing into pellets. Then, the samples were scanned using a Fourier infrared spectrometer (TENSOR27, Bruker Optics Co., Ltd., Bremen, Germany) with a scanning range of 4000 cm ^−1^ to 400 cm^−1^, accumulating 64 scans at a resolution of 4 cm^−1^. The KBr was used as the single-channel background reference. The secondary structure content of the protein samples was calculated using Omnic 8.0 and Peakfit 4.12 software.

### 2.13. Statistical Analysis

Data analysis was performed using SPSS v.26.0 (SPSS Inc., Chicago, IL, USA), and the normal distribution had been previously checked. The results are depicted as the mean ± standard deviation (SD). Analysis of variance (ANOVA) was used to assess the significance from two aspects, including different treatment groups and different storage times, and multiple analyses of means were conducted using the Duncan’s test at *p* < 0.05. A randomized block design, considering a mixed linear model including treatment and storage as fixed effects and manufacturing repetition as a random effect was used for analyzing all results. Each measurement was independently conducted three times, encompassing four different levels for each replication.

## 3. Results and Discussion

### 3.1. Changes in Moisture Content

**Figure 1:**
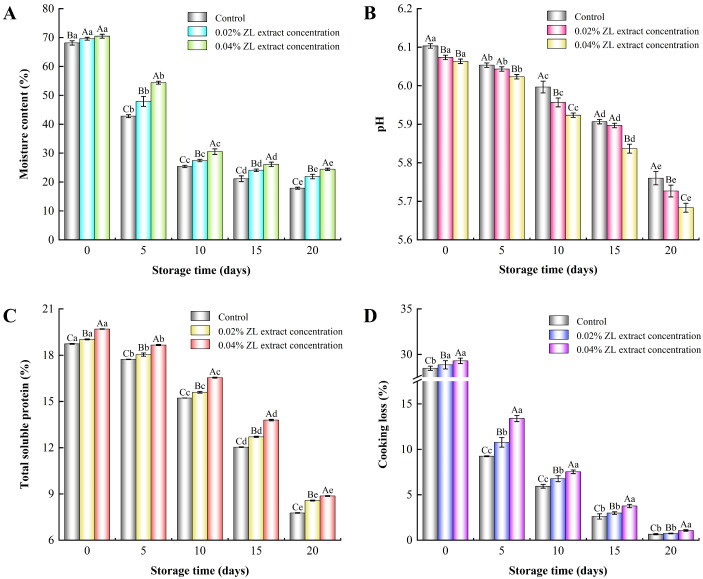
The enumeration of moisture content (**A**), pH (**B**), total soluble protein content (**C**), and cooking loss (**D**) in larou with different concentrations of ZL extract treatment during storage. Different capital letters (A–C) indicate significant differences on the same sampling time (*p* < 0.05); Different lowercase letters (a–e) indicate significant differences on the same batch (*p* < 0.05).

Water serves as a medium for biochemical reactions in cured meats, and moisture content is closely related to the texture and color of the cured meat products. The effect of ZL extract on the moisture content of the larou during storage is illustrated in Figure 1. The moisture content of all samples generally decreased with increased storage time, which could be associated with the water evaporation. The cross-linking of proteins during storage or a reduction in the hydrophilic properties of proteins due to oxidation were also other possible reasons [23]. Additionally, the gradual permeation of sodium chloride and sodium nitrite in larou also contributed to the decreased moisture content. At the initial stage (0 d) of storage, there was a significant difference in moisture content between the control group and the ZL extract-treated groups (*p* < 0.05), whereas no significant difference was observed between 0.02% and 0.04% ZL extract-treated groups (*p* > 0.05). This could be explained by the fact that ZL extract had water absorption capacity, relieving the loss of water content. After 20 d of storage, the moisture content in the control group decreased by 51.40%. However, the moisture content in the 0.02% and 0.04% ZL extract-treated groups decreased by 48.18% and 45.34%, respectively, indicating that the ZL extract could retard the water loss of larou during storage, which was probably attributed to the inhibitory effect of the ZL extract on protein oxidation [16]. Xu et al. [8] found similar results, in which *Cedrus deodara* extract improved the moisture content of larou. Moreover, it is reported that plant extracts can improve texture properties by alleviating moisture loss [24].

### 3.2. Changes in pH, Total Soluble Protein, and Cooking Loss of Larou

The change in pH value is an important indicator of meat product spoilage, and a decrease in pH value is beneficial to control the oxidation of fats and proteins, thereby inhibiting the production of off-flavors [5,25]. The effect of ZL extract on the pH value of larou is shown in Figure 1B. The pH value of the control and ZL extract-treated groups exhibited a decreasing tend as storage time increased. A decrease in the pH value of larou could lead to a reduction in the water-holding capacity of proteins [26], which is consistent with the results of the moisture content (Figure 1A). This phenomenon could be attributed to the acid production from microbial metabolism during storage [27]. On the same storage days (*p* < 0.05), the pH values of the ZL extract-treated groups were significantly lower than those of the control group, and the minimum values were observed in the 0.04% ZL extract-treated group. This result was similar to the findings of Wang et al. [28], where the addition of plant polyphenols could effectively reduce the pH value of dry-cured bacon during storage. A possible explanation for this was that ZL extract partially inhibited protein hydrolysis to the formation of alkaline substances such as ammonia and amines, leading to a decrease in the pH value of larou during storage [16]. On the other hand, a reduced pH value could inhibit the growth of spoilage microorganisms in larou, potentially serving to preserve the quality and safety of cured meat products.

The effect of ZL extracts on the total soluble protein content in larou is depicted in Figure 1C. As the storage time continued, there were general downward trends in the content of total soluble proteins in the control and ZL extract-treated groups, and the control group was distinctly lower than the ZL extract-treated groups. On the 20th day of storage, compared to samples on the 0th day of storage, the total soluble protein content of the control, 0.02%, and 0.04% ZL extract-treated groups decreased by 58.55%, 54.23%, and 52.68%, respectively, and the 0.04% ZL extract-treated group had the highest value (8.87%). This phenomenon could be attributed to the antioxidant effect of ZL extract, with the phenolic compounds of ZL extract producing hydrogen atoms through oxidation that combined with free radicals, thereby inhibiting the progression of oxidation, which resulted in a higher content of soluble proteins in ZL extract-treated groups compared to the control group during storage [16,29].

Cooking loss facilitates the discharge of intramuscular fluids, which are composed of water, soluble nutrients, and substances that contribute to the taste and flavor, along with pigments that are essential for coloration [30]. The effect of ZL extract on the cooking loss of larou during storage is illustrated in Figure 1D. As the storage time continued, the cooking loss of all samples generally showed a downward trend. From the 0th day to the 5th day, there is a significant reduction in the cooking loss of all samples; the control, 0.02%, and 0.04% ZL extract groups decreased by 19.24%, 18.07%, and 15.90%, respectively. These results are probably due to the fact that less moisture evaporated in the initial storage stages, and the ZL extract inhibited lipid oxidation and protein denaturation, thus maintaining the water retention of larou. Parafati, Restuccia, Palmeri, Fallico, and Arena [31] reported similar results, in which prickly pear extract improved the cooking loss of beef burger patties. However, at the end of 20th day of storage, less cooking loss was observed in all samples, attributed to the lower moisture content in larou [32].

### 3.3. Changes in Texture

The effect of ZL leaf extract on the texture properties of larou is shown in Table 1. As storage time increased, the hardness of the control group and ZL extract-treated groups showed a rising trend, which may be related to the exudation of lipids and moisture from the larou [33]. The hardness of the control group is significantly higher than that of the ZL extract-treated groups (*p* < 0.05), which may be attributed to the inhibitory effect of the ZL extract on protein oxidation in larou, leading to a more stable spatial structure of the proteins. This result was observed by Wu et al. [34], showing that the incorporation of catechin liposomes decreased the hardness of traditional Chinese bacon during storage. With the increase in storage time, the springiness and chewiness of the control group and the ZL extract-treated groups generally tended to rise, while the cohesiveness shows a decreasing trend. Meanwhile, no significance in springiness was obtained between the control group and ZL (*p* > 0.05), and the chewiness of the ZL extract-treated groups was always higher than that of the control group at the same storage times (*p* < 0.05). The 0.04% ZL extract-treated group exhibited notably higher chewiness than the 0.02% ZL extract-treated group (*p* < 0.05). The increase in chewiness of larou during storage may be due to the compactness of muscle fiber bundles induced by the decreased moisture [33]. Moreover, it has been reported that the interaction between phenolic compounds and myofibrillar protein was a crucial reason for the variation in the texture of meat products. Phenolic compounds can alter the structure of myofibrillar proteins, potentially causing the fragmentation of myofibrillar proteins and thereby improving the texture attributes of larou [8]. Our previous study reported that the ZL extract was rich in phenolic compounds, including chlorogenic acid, quercitrin, hyperoside, syringetin-3-glucoside, rutin, and vitexin [16]. Therefore, the incorporation of ZL extract could effectively improve the texture characteristics of larou during storage.

### 3.4. Changes in Instrumental Color

The effect of the ZL extract on the color of larou is shown in Table 2. The L* value of the control and ZL extract-treated groups gradually decreased with the storage time increase, and the control group exhibited peak values during the storage time, potentially due to the evaporation of surface moisture, which might reduce the transparency of the larou, leading to a decrease in the L* value. Similar results were reported by Wu et al. [34], where the L* value of traditional Chinese bacon gradually decreased with storage time. On the 20th day of storage, the L* values of the ZL extract-treated groups were remarkably lower than those of the control group, and 0.04% ZL extract-treated groups exhibited the valley value (34.75), which was in accordance with the findings of Xu et al. [8] on the impact of Cedrus deodara extract on the color changes in larou. A possible reason for this was that the ZL extract had an inherently dark color. The a* value of the control and ZL extract-treated groups declined significantly with increased storage time, which might be related to the further oxidation of myoglobin into reddish-brown metmyoglobin [35]. Another possible explanation is the interaction between myoglobin and aldehyde substances produced by lipid oxidation reactions, leading to a decrease in redness [36]. At the end of 20 days of storage, the a* value of the ZL extract-treated groups was significantly higher than that of the control group, and 0.04% ZL extract-treated group showed the peak value (2.81), which could be attributed to the inhibition of the oxidation of the ZL extract, contributing to the color-preserving effect [16]. The b* value of the cured meat is also associated with the degree of oxidation, with the higher b* value indicating greater oxidation. As storage time increases, there is a significant rise in the b* value of the control and ZL extract treated groups, which may be attributed to the continuous oxidation and denaturation of myoglobin during the storage process [36]. However, at the end of 20 days of storage, the b* value of the control group was significantly lower than that of the ZL extract-treated groups, which may be due to the inherent color of the ZL extract. Zhou et al. [24] reported similar results, in which plant extracts deceased the b* value of western-style smoked sausage. Furthermore, the ability of ZL extract to reduce the moisture content of larou was the another reason for the color changes, which is consistent with the variation in the results of the moisture content (Figure 1A).

### 3.5. Changes in TBARS

**Figure 2:**
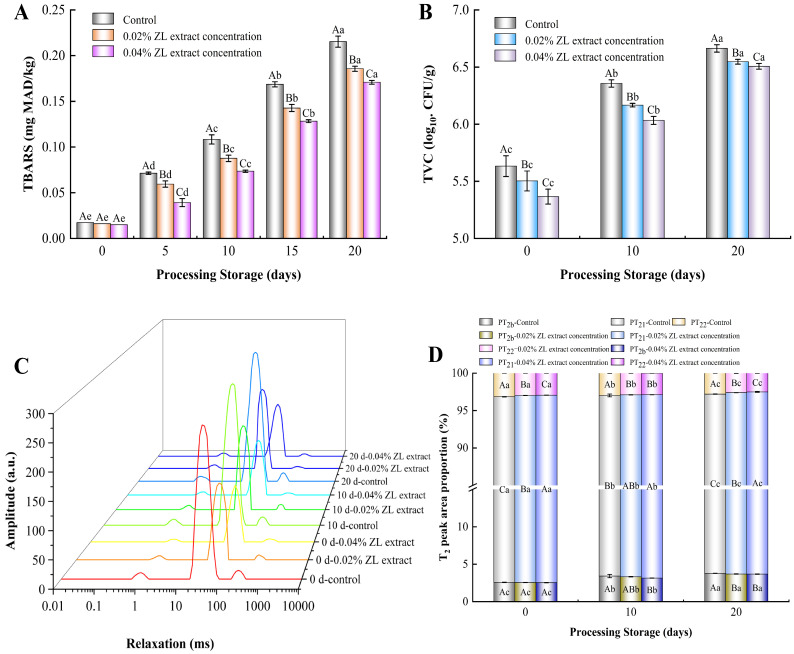
The enumeration of TBARS (**A**), TVC (**B**), the distribution of T_2_ relaxation times (**C**) and relaxation peak area proportions (**D**) in larou with different concentrations of ZL extract treatment during storage. Different capital letters (A–C) indicate significant differences on the same sampling time (*p* < 0.05); Different lowercase letters (a–e) indicate significant differences on the same batch (*p* < 0.05).

Changes in the TBARS values of the control and ZL extract groups are depicted in Figure 2A. During 20 days of storage, the TBARS values of all samples increased remarkably (*p* < 0.05), which was mainly due to lipid oxidation and the partial dehydration of larou during storage [18]. As the storage time continued, the control group exhibited higher TBARS values (0.07, 0.11, 0.17 and 0.22 mg MDA/kg on 5, 10, 15 and 20 days, respectively) than the ZL extract-treated groups. A previous study reported that natural plant extracts could exert an excellent effect on the partial inhibition of lipid oxidation during cured meat storage [5,8,34]. Therefore, the ZL extract-treated groups showed an inhibitory effect of lipid oxidation to a certain extent, and the 0.04% ZL extract-treated group showed minimum TBARS values throughout storage. In particular, at the end of the storage time (20th day), compared to the control (0.22 mg MDA/kg), the TBARS value (0.17 mg MDA/kg) of the 0.04% ZL extract-treated group was reduced by 22.73%. The antioxidant effect of the ZL extract could be explained by our previous study; the ZL extract is rich in polyphenolics such as chlorogenic acid, quercitrin, and hyperoside, which could scavenge free radicals or chelate metal ions to block the chain reaction of lipid oxidation in cured meats [37]. Furthermore, the inhibitory effect of polyphenolics in ZL extract on oxidative enzymes in larou was another possible explanation [15].

### 3.6. Changes in TVC

As depicted in Figure 2B, the TVC of all samples increased significantly during storage (*p* < 0.05), indicating that larou was a suitable nutrimental environment for the microorganism growth. However, on the same day during storage, the TVC of the ZL extract-treated groups was distinctly lower than that of the control group, and the TVC of the 0.04% ZL extract-treated group was lowest. At the end of storage (20th day), compared to the control group (6.65 log_10_ CFU/g), the TVC of the 0.04% ZL extract-treated group (6.51 log_10_ CFU/g) decreased by 5.67%, which was likely attributed to the antibacterial activity of the ZL extract, effectively inhibiting microbial growth during storage. Thus, the ZL extract decreased the microbial counts of larou during 20 days of storage. Similar findings were reported by Turan and Şimşek [38] and Wang et al. [5], where black mulberry extract improved the microbial quality of beef patties during storage and grape seed extract inhibited the microbial growth of smoke-cured bacon.

### 3.7. Changes in Low-Field NMR

The T_2_ relaxation times in larou are primarily divided into three peaks reflecting different water states, corresponding to bound water (T_2b_, 0–4 ms), immobilized water (T_21_, 14–160 ms), and free water (T_22_, 190–1200 ms). As shown in Figure 2C, the relaxation times of bound water, immobilized water, and free water in larou increased significantly with increased storage time. However, as the addition of ZL extract increased, the T_2_ relaxation time of larou gradually decreased and the relaxation time curves were altered, indicating that the incorporation of ZL extract affected the water distribution and migration of larou during storage. The study of Møller, Gunvig, and Bertram [39] revealed that water migration and changes in fermented sausages were intrinsically linked to the growth of microorganisms. Thus, the antibacterial effects of ZL extract could exert a substantial influence on the changes in the water distribution of larou. Additionally, the ZL extract addition increased the water binding ability of larou during storage, contributing to the shortened T_2_ relaxation times. Figure 2D presents the relative percentages of the different T_2_ components, which indicated that the ZL extract addition decreased the proportions of free water (PT_22_) and increased the proportions of immobilized water (PT_21_). Liu et al. [40] reported similar findings in which the incorporation of lysozyme, sodium alginate, and chitosan significantly increased the proportions of immobilized water and decreased the proportions of free water. A probable explanation for this is that the protein denaturation of larou during storage induced a reduction in water associated with proteins and large macromolecular components, while the antioxidant properties of ZL extract inhibited protein denaturation. On the other hand, the improvement of protein hydration by the interaction between the ZL extract and MP also contributed to these results. The change in low-field NMR results is crucial for understanding the water distribution and migration in meat products. Exploring these relaxation times can lead to a better understanding of the quality and preservation techniques of cured meat products.

### 3.8. Changes in Secondary Structures of MP

**Figure 3:**
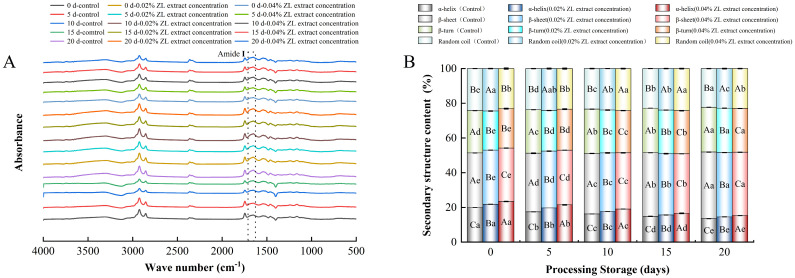
Enumeration of FT-IR (**A**) and percentages of secondary structural components (**B**) of myofibrillar protein in larou with different concentrations of ZL extract treatment during storage.

The amide I band (Figure 3A) between 1600 and 1700 cm^−1^, highly sensitive to hydrogen bonds and the main chains of polypeptides, is commonly used for analyzing the variation in the protein secondary structure. Wave numbers in the range of 1650–1660 cm^−1^ are typically associated with α-helices, those between 1610 and 1640 and 1670–1680 cm^−1^ with β-sheets, the regions of 1660–1670 and 1680–1700 cm^−1^ with β-turns, and the band at 1640–1650 cm^−1^ corresponds to the random coil conformation [41]. The variations in the secondary structures of MP in larou after ZL extract treatment during storage were shown in Figure 3B. As the storage time continued, the α-helix of the control and ZL extract-treated groups generally showed a downward trend and the α-helix of the control group decreased from 19.94% and 13.51% during storage for 20 days, which might be due to the lipid oxidation of larou and the reduction in water content, which disrupted the hydrogen bonds maintaining the α-helix structure [42]. At the end of storage (20th day), compared to the control (13.51%), the α-helix content of the 0.02% and 0.04% ZL extract-treated groups increased by 7.78% and 13.32%, respectively, probably because the ZL extract retarded the destruction of hydrogen bonds that maintained the α-helix structure by inhibiting lipid oxidation [43]. Contrary to the variation in α-helix results, the β-sheet contents of all samples increased significantly with the increased storage time (*p* < 0.05), which might be attributed to the protein oxidation of larou, leading to the transformation of an α-helix to β-sheet structure [13]. On the 20th day, compared to the control, the ZL extract-treated groups had remarkably lower β-sheet contents (*p* < 0.05) and the 0.04% ZL extract-treated group observed the minimum β-sheet content (36.48%). The interaction between MP and the ZL extract could inhibit the transformation of the α-helix to β-sheet structure, contributing to the decreased β-sheet content of larou. Thus, ZL extract treatment influenced the secondary structure of MP during larou storage. It was mentioned in the reports that natural plant extracts can interact with MP to improve the quality properties (such as the color and texture) of meat products by altering the spatial structure of MP [8]. Consequently, these results also further offered supplementary insights into the quality improvements observed in larou with the incorporation of ZL extract during storage.

## 4. Conclusions

In the present study, it was found that ZL extract can potently improve the physicochemical properties and storage qualities. The incorporation of ZL extract increased chewiness, a* value, b* value, total soluble protein content, and β-sheet content, and inhibited the increase in water loss, cooking loss, hardness, and TBARS value, which could be due to the antioxidant capacity of the ZL extract to prevent lipid and protein oxidation. Meanwhile, the L value, pH value, TVC, and α-helix content decreased with the addition of the ZL extract, possibly resulting from the antibacterial effect of the ZL extract and contributing to the quality improvement of larou during storage. The LF-NMR results further indicated that ZL extract addition promoted the conversion from free water to immobilized water during larou storage. Therefore, the aforementioned results demonstrated that the ZL extract held promise for enhancing the quality of cured meats and in the meat-processing field, and had potential and valuable application in the food industry. Future research should explore the impact of ZL extract on the potential correlation between free amino acids, volatile flavor compounds, and the microbial diversity of larou during storage at higher inclusion levels.

## Figures and Tables

**Table 1:** Enumeration of texture in larou with different concentrations of ZL extract treatment during storage.

Concentration of ZL Extract	Storage Time (Days)				
	0	5	10	15	20
Hardness (kg)					
0	2.85 ± 0.12 ^Ae^	8.80 ± 0.12 ^Ad^	18.30 ± 0.14 ^Ac^	24.94 ± 0.01 ^Ab^	34.56 ± 0.25 ^Aa^
0.02%	2.33 ± 0.05 ^Be^	7.40 ± 0.07 ^Bd^	14.09 ± 0.20 ^Bc^	23.41 ± 0.63 ^Bb^	30.66 ± 0.29 ^Ba^
0.04%	2.14 ± 0.12 ^Be^	5.65 ± 0.01 ^Cd^	11.90 ± 0.22 ^Cc^	21.53 ± 0.19 ^Cb^	28.72 ± 0.13 ^Ca^
Springiness (mm)					
0	0.45 ± 0.02 ^Ae^	0.49 ± 0.01 ^Ad^	0.54 ± 0.01 ^Ac^	0.59 ± 0.01 ^Bb^	0.68 ± 0.02 ^Aa^
0.02%	0.43 ± 0.00 ^Ae^	0.47 ± 0.02 ^Ad^	0.57 ± 0.02 ^Ac^	0.64 ± 0.03 ^Ab^	0.67 ± 0.02 ^Aa^
0.04%	0.45 ± 0.02 ^Ad^	0.49 ± 0.01 ^Ac^	0.58 ± 0.03 ^Ab^	0.63 ± 0.01 ^Aa^	0.65 ± 0.01 ^Aa^
Cohesiveness					
0	0.55 ± 0.01 ^Aa^	0.49 ± 0.02 ^Ab^	0.44 ± 0.01 ^Ac^	0.30 ± 0.03 ^Bd^	0.28 ± 0.01 ^Ad^
0.02%	0.55 ± 0.01 ^Aa^	0.44 ± 0.02 ^Bb^	0.4 ± 0.02 ^Bbc^	0.38 ± 0.02 ^Ac^	0.31 ± 0.04 ^ABd^
0.04%	0.57 ± 0.04 ^Aa^	0.47 ± 0.03 ^ABb^	0.42 ± 0.01 ^ABbc^	0.39 ± 0.00 ^Acd^	0.36 ± 0.03 ^Ad^
Chewiness (N.mm)					
0	0.54 ± 0.04 ^Be^	1.34 ± 0.04 ^Cd^	2.79 ± 0.06 ^Cc^	3.86 ± 0.45 ^Bb^	5.41 ± 0.21 ^Ba^
0.02%	0.55 ± 0.01 ^Be^	1.53 ± 0.06 ^Bd^	3.19 ± 0.09 ^Bc^	5.56 ± 0.21 ^Ab^	6.36 ± 0.76 ^Ba^
0.04%	0.73 ± 0.02 ^Ae^	2.01 ± 0.10 ^Ad^	4.45 ± 0.13 ^Ac^	6.09 ± 0.09 ^Ab^	8.08 ± 0.65 ^Aa^

Note: Values with different capital letter letters (A–C) at the same sampling time were significantly different (*p* < 0.05); Values with different lowercase letters (a–e) in the same batch were significantly different (*p* < 0.05).

**Table 2:** Enumeration of color in larou with different concentrations of ZL extract treatment during storage.

Concentration of ZL Extract	Storage Time (Days)				
	0	5	10	15	20
L*					
0	44.68 ± 0.49 ^Aa^	42.62 ± 0.10 ^Ab^	40.43 ± 0.13 ^Ac^	37.91 ± 0.03 ^Ad^	36.46 ± 0.13 ^Ae^
0.02%	43.89 ± 0.04 ^Ba^	41.53 ± 0.05 ^Bb^	39.74 ± 0.17 ^Bc^	37.02 ± 0.19 ^Bd^	35.72 ± 0.04 ^Be^
0.04%	43.73 ± 0.03 ^Ba^	40.96 ± 0.06 ^Cb^	38.94 ± 0.06 ^Cc^	36.05 ± 0.10 ^Cd^	34.75 ± 0.16 ^Ce^
a*					
0	4.30 ± 0.01 ^Ca^	3.79 ± 0.09 ^Cb^	3.50 ± 0.01 ^Cc^	3.03 ± 0.05 ^Cd^	2.46 ± 0.02 ^Ce^
0.02%	4.47 ± 0.03 ^Ba^	4.00 ± 0.03 ^Bb^	3.62 ± 0.02 ^Bc^	3.16 ± 0.02 ^Bd^	2.57 ± 0.02 ^Be^
0.04%	4.64 ± 0.03 ^Aa^	4.13 ± 0.05 ^Ab^	3.69 ± 0.01 ^Ac^	3.27 ± 0.01 ^Ad^	2.81 ± 0.03 ^Ae^
b*					
0	3.08 ± 0.01 ^Ce^	3.34 ± 0.02 ^Cd^	3.76 ± 0.02 ^Cc^	4.23 ± 0.05 ^Cb^	5.12 ± 0.03 ^Ba^
0.02%	3.13 ± 0.01 ^Be^	3.48 ± 0.01 ^Bd^	3.97 ± 0.02 ^Bc^	4.38 ± 0.01 ^Bb^	5.17 ± 0.02 ^ABa^
0.04%	3.24 ± 0.01 ^Ae^	3.55 ± 0.02 ^Ad^	4.05 ± 0.02 ^Ac^	4.62 ± 0.02 ^Ab^	5.22 ± 0.03 ^Aa^

Note: Values with different capital letter letters (A–C) at the same sampling time were significantly different (*p* < 0.05); Values with different lowercase letters (a–e) in the same batch were significantly different (*p* < 0.05).

## Data Availability

The original contributions presented in this study are included in the article. Further inquiries can be directed to the corresponding author.
